# Latent profile analysis of mindful self-care and its relationship with professional grief among intensive care unit nurses

**DOI:** 10.3389/fpubh.2026.1948775

**Published:** 2026-09-25

**Authors:** Xingru Chen, Lixin Kang, Yuxiao He, Huiping Zhang, Dongping Zhang, Pingping Mu, Xiaomin Zhao

**Affiliations:** 1 School of Nursing, Shandong Medical and Pharmaceutical University, Yantai, Shandong, China; 2 Qilu Hospital of Shandong University Dezhou Hospital, Dezhou, Shandong, China; 3 Department of Anesthesiology, The Affiliated Yantai Yuhuangding Hospital of Qingdao University, Yantai, Shandong, China; 4 Department of Critical Care Medicine, Yantai Affiliated Hospital of Shandong Medical and Pharmaceutical University, Yantai, Shandong, China

**Keywords:** influencing factors, intensive care unit nurse, latent profile analysis, mindful self-care, professional grief

## Abstract

**Introduction:**

Intensive Care Unit (ICU) nurses are particularly at a high risk of professional grief. Mindful self-care practices were confirmed to improve nurses' mental health status. However, the current studies focusing on mindful self-care among ICU nurses are limited, and the relationship between mindful self-care and professional grief has not been confirmed. This study aims to examine the characteristics and influencing factors of different mindful self-care latent profiles among ICU nurses and investigate the relationship between mindful self-care and professional grief.

**Methods:**

The survey was conducted on 355 ICU nurses from nine hospitals in Shandong Province using the General Demographic Questionnaire, Mindful Self-Care Scale, Grief State Scale Nurses, and Short Form of the Self-Compassion Scale. Latent profile analysis was used to determine the profiles of mindful self-care among ICU nurses. Binary logistic regression was employed to explore the influencing factors of different profiles. A *t*-test was used to compare professional grief differences among distinct mindful self-care profiles.

**Results:**

Mindful self-care among ICU nurses was categorized into the “mindful self-care deficiency profile” (32.5%) and “high mindfulness care profile” (67.5%). Factors influencing mindful self-care profiles included health status, and self-compassion ability (*p* < 0.05). High mindfulness care profile nurses exhibited lower levels of professional grief (*p* < 0.001).

**Conclusions:**

This study classified ICU nurses' mindful self-care into two latent profiles. ICU nurses with good health status and high self-compassion were more likely to belong to the high-mindfulness-care profile, whose members reported lower professional grief. These findings provide evidence for interventions targeting mindful self-care and professional grief among ICU nurses.

## 1 Introduction

Intensive Care Unit is particularly characterized by high workloads, complex techniques, and a high concentration of critically ill patients. ICU nurses are required to provide high-quality care under stringent working conditions, frequently dealing with emergencies and life-and-death situations (1). However, the prolonged heavy workload and emotional overexertion lead to continuous work stress and the risk of professional grief. Professional grief is a negative psychological state resulting from handling patients' suffering, deterioration, and death frequently (2). Recent research showed that ICU nurses generally feel grief when patients die, and the prolonged professional grief experience can impose psychological and emotional burdens, exacerbating the occurrence of burnout and compassion fatigue (3). Research suggests that the impact of professional grief on nurses is related to their ability to adapt and regulate emotions, but ICU nurses often lack adaptive coping strategies to counter this grief (3). Over time, unresolved grief can accumulate into excessive professional grief, negatively impacting both personal wellbeing and professional development. ICU nurses need to find relevant factors to promote self-emotional regulation and mitigate professional grief urgently.

Mindfulness refers to using intentional, non-judgmental, and present-moment-focused awareness ways to perceive and accept one's internal and external experiences. It facilitates individuals in mitigating excessive preoccupation with past regrets or future anxieties and enhances their ability to focus on current sensations and behaviors (4). Mindfulness-based interventions have been proven beneficial for fostering healthier lifestyle patterns and improving psychological wellbeing (5, 6). Additionally, research indicated that enhancing self-care competence is crucial for improving nurses' health status and promoting professional development (7). However, traditional self-care is often viewed as a task rather than a healthy lifestyle, and over 90% of healthcare professionals lack self-care plans, neglecting their own health (8). Self-compassion represents a core inner psychological resource closely intertwined with mindful self-care. Theoretical and empirical works indicated that self-compassion is an important inner prerequisite for nurses to implement mindful self-care behaviors (9). Cook-Cottone integrated mindfulness with the self-regulation theory of traditional self-care to propose the concept of mindful self-care based on the attuned representational model of self (ARMS) (10). ARMS conceptualizes the self as an integrative system in which internal experiences, including physiological, emotional, and cognitive processes, interact dynamically with external contexts such as family, community, and culture (11). Within this framework, mindful self-care entails consciously appraising work-life demands and intentionally engaging in self-care practices (11). These proactive practices may help meet psychological needs and maintain a balance between internal resources and external demands. For ICU nurses, repeated exposure to patient death may generate negative emotions that remain inadequately processed in the absence of adaptive self-care, thereby increasing vulnerability to professional grief. Conversely, adequate mindful self-care may support emotional processing and buffer against the adverse psychological effects associated with such occupational exposure.

Research indicates that continuous engagement in mindful self-care practices can improve nurses' mental health, alleviate negative emotions and compassion fatigue, and enhance the quality of professional life (12, 13). For instance, a study by Zhang et al. (14) on oncology nurses revealed that nurses with higher levels of mindful self-care were more proactive in seeking internal and external support to cope with challenges, allowing them to maintain emotional balance in the face of grief. Studies by Hotchkiss (15) and Garcia et al. (16) found that mindful self-care can increase compassion satisfaction among hospice workers, alleviate compassion fatigue, and improve the quality of professional life. Additionally, engaging in mindful self-care practices can enhance nurses' resilience, reduce the risk of burnout, and improve work efficiency. For example, research by Craigie et al. (17) found that mindful self-care practice can effectively reduce occupational burnout and stress among nurses, thereby improving work efficiency. Slatyer et al. (18) used a randomized controlled trial and found that compared to the control group, the intervention group of nurses showed significantly reduced burnout and depressive symptoms after completing mindful self-care interventions. Therefore, enhancing ICU nurses' mindful self-care competence is significant for emotional regulation and stress management to mitigate their psychological burden.

Current cross-sectional studies tend to focus on the value of mindful self-care, without thoroughly analyzing its influencing factors. Some influencing factors also remain controversial and require further exploration. This may affect the construction of intervention-targeted measures. For example, Zeb et al. (19) found that female nurses exhibit higher levels of mindful self-care than male nurses, while Ahmadi et al. (20) reported no statistically significant differences between the genders. The relationship between gender and mindful self-care thus warrants further investigation. The research by Zeb et al. (19) also indicated that the level of mindful self-care is negatively correlated with age and years of nursing work experience. They consider that as nurses age and gain more experience, their stress and burnout levels may increase, negatively affecting their mindful self-care practices. This conclusion aligns with the research of Chipu et al. (21) but contradicts the view of Garcia et al. (16) that older nurses with more experience are better equipped to engage in introspective and reflective practices, leading to higher mindful self-care levels. Zeb et al. (19) also found that nurses with higher educational levels exhibited low mindful self-care levels. This finding is opposite to Zhang et al. (14) who reported a positive correlation between education and mindful self-care.

Notably, current research on the status and influencing factors of mindful self-care among nurses predominantly adopts a variable-centered approach. This approach assesses the overall level of mindful self-care and its influencing factors based on scale scores, often neglecting individual differences. This oversight may lead to a lack of specificity in intervention program development, thereby affecting the implementation of mindful self-care intervention studies. Latent Profile Analysis (LPA) is a person-centered research method that emphasizes heterogeneity within the sample (22). It can identify latent characteristic classifications based on individual differences, thereby providing targeted intervention measures according to these classifications (23). Chinese scholars Shi et al. (24) and A et al. (25) have separately used LPA to categorize the mindful self-care level among oncology and emergency nurses into three profiles: high, moderate, and low. These researches provide references to explore the mindful self-care level further using a person-centered approach. Therefore, considering the limitation of current research on ICU nurses' mindful self-care and previous studies lacked correlations between mindful self-care and professional grief. This study aims to (1) explore the different latent profiles of mindful self-care among ICU nurses, (2) analyze the influencing factors of different profiles, and (3) investigate the relationship between different mindful self-care latent profiles and professional grief. This will provide new bases for improving mindful self-care and mitigating professional grief among ICU nurses.

## Materials and methods

### Study design

This was a descriptive cross-sectional study that examined the characteristics and influencing factors of different mindful self-care latent profiles among ICU nurses and investigated the relationship between mindful self-care and professional grief.

### Participants

The cluster sampling method was used to survey ICU nurses of nine hospitals in Shandong Province from January to February 2024. The inclusion criteria were nurses who were in-service registered nurses working in Intensive Care Unit and gave their informed consent and voluntarily participated in the research. The exclusion criteria were nurses who were receiving full-time further education outside the hospital (completely detached from routine ICU clinical work during data collection); on long-term leave and temporarily disengaged from ICU clinical practice.

The sample size for this study was calculated based on the mindful self-care scores. We use the formula ($n=\frac{U_{\frac{\alpha }{2}}^{2}\sigma^{2}}{\delta^{2}}$) to estimate sample size. Based on previous literature, assuming α = 0.05, σ = 13.91 (25), δ = 1.5, and a non-response rate of 10%, the calculated sample size was 368, which can meet the needs of LPA (26) and binary logistic regression analysis. A total of 385 questionnaires were distributed, of which 355 valid questionnaires were returned and included in the analysis. The effective response rate was 92.2%. The study was conducted according to the guidelines of the Declaration of Helsinki, and approved by the Institutional Review Board of Binzhou Medical University (IRB: 2024-L002).

### Instruments

#### The general demographic questionnaire

The demographic questionnaire was conducted based on previous literature (14). This scale comprised six items, including gender, age, professional title, years of working, education level, and self-rated health status.

#### The brief mindful self-care scale (B-MSCS)

The B-MSCS was developed by Hotchkiss and Cook-Cottone (27) and translated into a Chinese version by Yang et al. (28). This scale includes mindful awareness, mindful relaxation, physical care, self-compassion and purpose, supportive relationships, and supportive structures six dimensions, with a total of 24 items. Items are scored using Likert's 5-level rating system (ranging from 1 = completely non-compliant to 5 = completely compliant). A higher score indicates a greater level of Mindful Self-Care. The overall Cronbach's alpha (α) of the scale was 0.920 (29). In this study, the Cronbach's alpha (α) for this scale was 0.930.

#### The short form of the self-compassion scale (SCS-SF)

The SCS-SF was developed by Raes et al. (29). This scale includes self-kindness, self-judgment, common humanity, isolation, mindfulness, and overidentification six dimensions, with a total of 12 items. Items are scored using Likert's 5-level rating system (ranging from 1 = completely non-compliant to 5 = completely compliant). The overall Cronbach's alpha (α) of the scale was 0.870. In this study, the Cronbach's alpha (α) for this scale was 0.868.

#### The grief state scale for nurses (GSSN)

The GSSN was developed by Betriana et al. (2) and translated into a Chinese version by Wang et al. (30), including grief traits and grief state two subscales. It comprises four dimensions, including emotional mood swings, uncomfortable feelings toward death, feeling sadness toward patient death, and emotional exhaustion after caring, with a total of 17 items. Items are scored using Likert's 5-level rating system (ranging from 1 = completely non-compliant to 5 = completely compliant). The overall Cronbach's alpha (α) of the scale was 0.813. In this study, the Cronbach's alpha (α) for this scale was 0.932.

### Data collection

The questionnaire was designed using the Chinese survey website Wenjuanxing platform (www.sojump.com). After approval from the nursing department, we appointed a research leader in each hospital who distributed the questionnaire link to the WeChat groups of each hospital. Nurses provided informed consent and completed the questionnaire anonymously, with each WeChat ID permitted to submit only once. After collecting questionnaires, predefined screening criteria were applied to identify invalid responses. Questionnaires were excluded if they contained logical inconsistencies (e.g., contradictory answers to mutually exclusive items) or demonstrated straightlining responses (identical Likert-scale responses across all scale items). In total, 14 questionnaires were excluded due to straightlining, and 16 questionnaires were excluded due to logical inconsistency.

### Statistical analysis

LPA was conducted using Mplus V.8.3 (Muthén & Muthén, Los Angeles, CA, USA) software (31). Latent profile analysis was performed using maximum likelihood with robust standard errors (MLR) estimator; default convergence criteria and 500 random starting sets were applied to avoid local maxima. The initial model was iteratively adjusted by increasing the number of profiles until the optimal model was identified based on the fitting index. The fitting index for the latent profile model includes the Akaike Information Criterion (AIC), the Bayesian Information Criterion (BIC), the Sample-Adjusted Bayesian Information Criterion (aBIC), Entropy, the Lo-Mendell-Rubin Likelihood Ratio Test (LMR), and the Bootstrap Likelihood Ratio Test (BLRT). Smaller values of AIC, BIC, and aBIC indicate better model fit. Entropy ≥ 0.8 suggests classification accuracy exceeding 90%. Statistical significance (*p* < 0.05) in the LMR and BLRT indicates the superiority of the K profiles model over the K-1 profiles model (31). Statistical analysis was performed using IBM SPSS 25.0 (IBM Corporation, Armonk, NY, USA). Frequencies, percentages, means, and standard deviations (SD) were used to describe the participants' demographic and work-related variables and mindful self-care. Group differences in categorical variables were assessed using the chi-square test or Fisher's exact probability method (group differences in categorical variables were assessed using the chi-square test; Fisher's exact probability method was applied when more than one cell had expected frequencies < 5), while continuous variables were analyzed with the independent samples *t*-test. Binary logistic regression analysis was used to analyze the influencing factors, the fitted profile was used as the dependent variable, and the demographic and work-related variables were used as the independent variables. The significance level was set at α = 0.05 (two-tailed).

## Results

### Participant characteristics

Among the 355 surveyed nurses, 53 were male (14.9%), and 302 were female (85.1%). Participants ages ranged from 20 to 49 years, with an average age of 31.39 years. A total of 43 nurses had an associate degree (12.1%), 299 had a bachelor's degree (84.2%) and 13 had a master's degree or higher (3.7%).

### Common method bias test

Harman's single-factor test was used to assess common method bias across all scale items (32). Ten factors with eigenvalues >1 were extracted. The variance explained by the first factor was 26.84%, which is below the 40% critical threshold, indicating that common method bias is not significant in this study.

### Latent profile analysis results of mindful self-care among ICU nurses

Table 1 displays the fitting index of the LPA model. According to the scores of the six dimensions of the Mindful Self-Care Scale, the profiles of mindful self-care were divided into one to four profiles by the LPA method. Results showed that AIC, BIC, and aBIC decreased with an increasing number of profiles. However, the LMR became non-significant (*p* > 0.05) in the three-profile model, suggesting a better fit for the two-profile model than the three-profile model. The values of AIC, BIC, and aBIC in the two-profile model were smaller, and the entropy value of this model was >0.80, indicating higher classification accuracy. Therefore, the two-profile classification model was selected as the optimal model.

**Table 1:** Fitting index for latent profiles of mindful self-care among ICU nurses (*N* = 355).

Number of profiles	AIC	BIC	aBIC	LMRT	BLRT	Entropy	Class probability
1	4,906.003	4,952.469	4,914.400	—	—	—	
2	4,350.990	4,424.561	4,364.284	0.001	< 0.001	0.873	0.325/0.675
3	3,907.991	4,008.666	3,926.183	0.196	< 0.001	0.956	0.264/0.583/0.153
4	3,500.361	3,628.141	3,523.451	0.111	< 0.001	0.997	0.248/0.031/0.553/0.168

AIC, Akaike Information Criterion; BIC, Bayesian Information Criterion; aBIC, Sample-Adjusted Bayesian Information Criterion; LMR, Lo–Mendell–Rubin likelihood ratio test; BLRT, Bootstrap Likelihood Ratio Test.

### Latent profiles and nomenclature of mindful self-care among ICU nurses

Figure 1 displays the profiles of mindful self-care among ICU nurses and the scores of the two profiles on different dimensions of the mindful self-care Scale. Profile 1 comprising 112 individuals (32.5% of the total), scored low across all six dimensions of mindful self-care. This suggested that nurses in this profile have inadequate mindfulness care and physical self-care abilities. Therefore, this profile was named “Mindful Self-Care Deficient Profile.” Profile 2 consisting of 243 individuals (67.4% of the total), scored high in dimensions such as mindful awareness, mindful relaxation, self-compassion and purpose, supportive relationships, and supportive structures but scored low in physical care. This indicated that while nurses in this profile exhibit high levels of mindfulness care, their physical self-care needed improvement. Thus, this profile was named “High Mindfulness Care Profile.”

**Figure 1:**
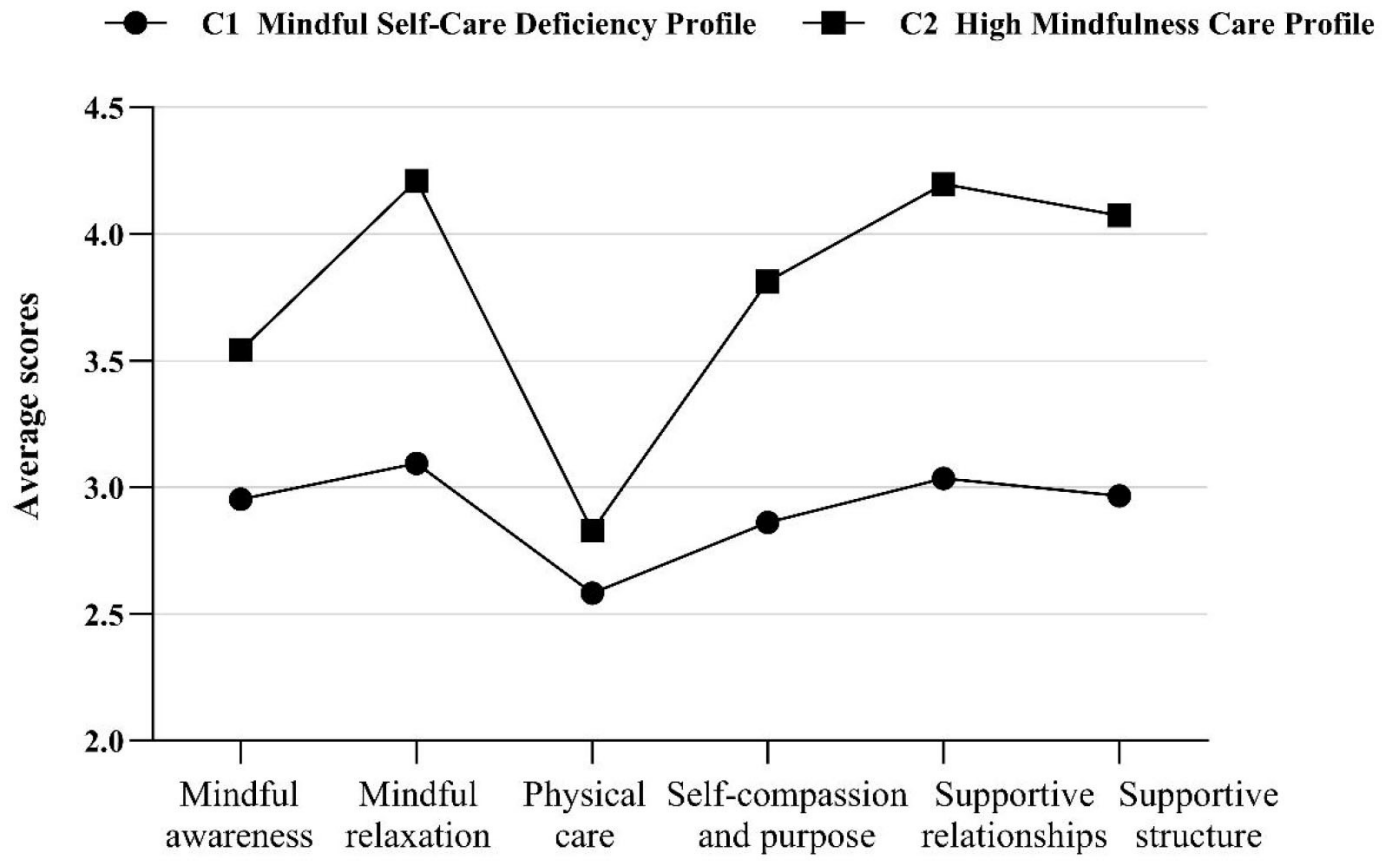
The scoring of mindful self-care latent profiles among ICU nurses.

### Demographic characteristics of different profiles of mindful self-care among ICU nurses

Table 2 displays the demographic characteristics among ICU nurses with different mindful self-care latent profiles. The results revealed significant differences in education level, self-rated health status, and self-compassion abilities between the two mindful self-care profiles among ICU nurses (*p* < 0.05).

**Table 2:** Comparison of demographic characteristics of ICU nurses with different mindful self-care latent profiles (*N* = 355).

Item	Mindful self-care deficient profile (*N* = 112)	High mindfulness care profile (*N* = 243)	Test statistic	*p*-Value
Gender			2.862^a^	0.091
Male	22 (19.6%)	31 (12.8%)		
Female	90 (80.4%)	212 (87.2%)		
Age			2.528^a^	0.112
20–30 years	59 (52.7%)	106 (43.6%)		
>30 years	53 (47.3%)	137 (56.4%)		
Professional title			5.658^a^	0.059
Nurse	21 (18.8%)	53 (21.8%)		
Senior nurse	47 (42.0%)	71 (29.2%)		
Chief nurse and above	44 (39.2%)	119 (49.0%)		
Education level			—	0.014
Associate degree or below	17 (15.2%)	26 (10.7%)		
Bachelor's degree	95 (84.8%)	204 (84.0%)		
Master's degree or above	0 (0.0%)	13 (5.3%)		
Years of working			4.023^a^	0.259
≤ 4	37 (33.1%)	84 (34.6%)		
5–9	39 (34.8%)	61 (25.1%)		
10–14	26 (23.2%)	69 (28.4%)		
≥15	10 (8.9%)	29 (11.9%)		
Self-rated health status			32.935^a^	< 0.001
Poor	24 (21.4%)	13 (5.3%)		
Average	67 (59.8%)	125 (51.4%)		
Good	21 (18.8%)	105 (43.3%)		
Self-compassion scores	35.17 ± 3.04	38.27 ± 3.92	7.277^b^	< 0.001

^a^Represents the value of χ^2^.

^b^Represents the t-value.

—Represents Fisher's exact probability method results.

### Binary logistic regression analysis of latent profiles in mindful self-care

Table 3 displays the results of binary logistic regression analysis of factors influencing the latent profiles of mindful self-care. Binary logistic regression analysis was performed with the two latent profiles of mindful self-care as the dependent variable (coded as Deficient Mindful Care Profile = 0, High Mindfulness Care Profile = 1) and the variables with significant differences in the univariate analysis were the independent variables. The results revealed that ICU nurses in average or good health status, with high self-compassion were more likely to belong to the “High Mindfulness Care Profile” (*OR* = 2.687, 6.056, 1.325). Model diagnostics indicate no significant multicollinearity (all VIF < 5); the Hosmer–Lemeshow test suggests acceptable model fit (χ^2^ = 13.885, *p* = 0.053), with Nagelkerke *R*^2^ = 0.294.

**Table 3:** Binary logistic regression analysis of factors influencing the latent profiles of mindful self-care among ICU nurses (*N* = 355).

Variable	β	OR (95% CI)	Wald χ^2^	*p*-Value
Self-rated health status (reference: poor)				
Average	0.393	2.687 (1.244, 5.802)	6.330	0.012
Good	0.439	6.056 (2.561, 14.320)	16.827	< 0.001
Self-compassion	0.055	1.325 (1.190, 1.475)	26.228	< 0.001

### Relationship between latent profiles of mindful self-care and professional grief

Table 4 displays differences in total and dimensional professional grief scores among ICU nurses with different latent profiles. Significant differences were observed in all dimensions except for feeling sadness toward patient deaths (*p* < 0.05).

**Table 4:** Comparison of professional grief scores among ICU nurses with different latent profiles (*N* = 355).

Professional grief scores	Mindful self-care deficient profile (*N* = 112)	High mindfulness care profile (*N* = 243)	*t-*Value	*p*-Value
Total professional grief scores	38.46 ± 12.16	33.73 ± 10.41	3.773	< 0.001
Emotional mood swings	9.69 ± 3.91	7.37 ± 3.42	5.672	< 0.001
Uncomfortable feelings toward deaths	6.37 ± 2.79	5.67 ± 2.68	2.226	0.027
Feeling sadness toward patient deaths	12.62 ± 4.10	12.34 ± 3.65	0.643	0.521
Emotional exhaustion after caring	9.79 ± 3.97	8.35 ± 3.59	3.409	0.001

## Discussion

In this research, we use a person-centered approach to identify the level of mindful self-care among ICU nurses. This study identified two distinct latent profiles of mindful self-care, then examined factors affecting the latent profiles, and finally explored the relationship between mindful self-care profiles and professional grief. As far as we know, this research is the first to explore mindful self-care and professional grief status among ICU nurses through person-centered research. These findings can provide clues for formulating targeted interventions to improve ICU nurses' mindful self-care and professional grief levels.

This study employed LPA to categorize mindful self-care into “Mindful Self-Care Deficient Profile” and “High Mindfulness Care Profile” two profiles. Among the 355 individuals, the “Mindful Self-Care Deficient Profile” accounted for 32.5% of the sample, scoring low in all dimensions. This indicates that about one-third of ICU nurses struggle to balance internal and external demands, lack conscious assessment and fulfillment of their needs, and exhibit poor self-care behaviors. ICU nursing places high requirements on technical and specialty competence (33). These nurses usually face serious transition shock (34), which may result in increased stress and pressure, particularly for nurses with lower educational backgrounds. This stress may hinder their ability to balance work demands and self-care, resulting in low mindful self-care. The “High Mindfulness Care Profile” accounts for 67.4% of the sample, scoring high in all dimensions except physical care. This indicates that while most ICU nurses demonstrate a high level of mindfulness care, they still have deficiencies in physical self-care practices, such as diet and exercise. This profile is characterized by nurses with a master's degree and good health status. These nurses may be more likely to engage in positive self-care-related behaviors. Existing evidence indicates that the better the self-reported health status of clinical nurses, the higher the degree of participation in health promotion behaviors, and the better the mindful self-care behaviors (14). We found an interesting finding that both latent profiles scored low on the physical-care dimension despite overall score heterogeneity, This phenomenon that can be explained by the high-demanding nature of ICU nursing. Since critically ill patients require round-the-clock monitoring, ICU nurses often have short, irregular meal schedules that undermine regular nutritional intake. Furthermore, the heavy workload consumes substantial physical and mental resources, leaving nurses with limited energy and motivation for personal physical exercise after work (35).

This study found health status, and self-compassion were important factors impacting mindful self-care profiles. First, nurses with good health status are more likely to develop a high mindfulness care profile, consistent with research conducted by Zhang et al. (14). Nurses with good health have a clearer understanding of their emotional states and physical sensations, making it easier to recognize their needs. They are more inclined to adopt healthy lifestyles, motivated and capable of learning and practicing mindfulness techniques, and actively engaging in mindful self-care practices. Second, nurses with high self-compassion abilities are more likely to develop a high mindfulness care profile, which is consistent with the results of Zhang et al. (14). High self-compassion promotes nurses' care and attention to themselves, enhancing awareness and respect for their physical and mental needs, and fostering behaviors and habits that maintain health (14). Moreover, nurses with higher self-compassion levels are more proactive in seeking internal and external help, better understanding and accepting work challenges, and adopting positive strategies to cope with stress, thereby actively implementing mindful self-care.

Furthermore, the study found nurses with a high mindfulness care profile exhibited lower levels of professional grief (*p* < 0.001). Mindful self-care belonging to the domain of positive psychology, includes internal self-practice, external self-practice, and integrative practice three practice types (36). Internal self-practice enhances physical strength and fosters a growth mindset to face failures and challenges. ICU nurses often encounter situations of deteriorating patient conditions and life-and-death scenarios, which significantly impact their psychological state. High mindfulness care profile nurses can develop psychological resilience to cope with high-intensity work and emotional shocks, reducing grief caused by work. External self-practice provides a stable support system to alleviate work-related psychological stress. High mindfulness care profile nurses have good supportive relationships to offer emotional support and understanding, making them feel less isolated when facing work pressures. Meanwhile, a stable support structure provides a comfortable and organized living and working environment, helping nurses maintain a positive mindset and stable emotions, reducing the occurrence of professional grief. Integrative practice helps individuals recognize the importance of self-care and enhances their motivation for self-care. The high mindfulness care profile nurses have higher mindfulness awareness and richer mindfulness techniques. They are better at accepting and focusing on their current internal experiences, using mindfulness techniques to cope with emotions and stress. This flexibility in handling emotional responses helps them avoid over-involvement in patients' emotions, thereby reducing the risk of professional grief. Currently, mindful self-care and resiliency interventions (MSCR) has been shown to reduce nurses' stress and anxiety, enhancing emotional wellbeing (17). Nursing managers can implement similar programs among ICU nurses to improve their mindful self-care levels and further reduce their professional grief.

Notably, no significant inter-group difference was observed for the dimension “feeling sadness toward patient deaths” (*p* = 0.521). This may be sadness triggered by patient death represents a normal, universal emotional response for ICU nurses facing end-of-life patients, Mindful self-care may mitigate secondary negative outcomes including emotional exhaustion, mood swings, and death-related discomfort, but it cannot completely eliminate the primary sadness elicited by witnessing patient death (3).

Based on the above findings, we propose several recommendations to enhance mindful self-care among ICU nurses. First, nursing managers can provide appropriate training and support to help nurses master equipment and nursing work routines, offering recognition and incentives to boost work confidence and reduce stress. Managers may also enhance mindfulness and self-care training, helping young nurses learn mindfulness techniques to regulate emotions and cope with stress (4), thereby improving nurses' mindful self-care abilities. Second, nursing managers should formulate reasonable shift schedules, create a supportive working environment, and improve mental health protection. Moreover, the hospital's nutrition department can offer diverse meal delivery services to improve the nutritional and health conditions of nurses. Previous studies indicated that aerobic exercise and muscle training can effectively improve nurses' health status (37). Thus, nursing managers also can encourage nurses to strengthen physical exercise and adopt healthy lifestyles to enhance mindful self-care (38). Finally, given that self-compassion can positively impact mindful self-care levels, managers can improve nurses' mindful self-care abilities through a mindful self-compassion program (39).

This study has several limitations. First, it only selected nurses from hospitals in Shandong Province for a cross-sectional survey. This may limit data generalizability, restricting a comprehensive description of the mindful self-care level among ICU nurses nationwide. Second, all variables in this study were measured using self-report questionnaires, which may lead to response bias. Third, based on Monte-Carlo simulation evidence for latent profile analysis (LPA) (40), our sample size of 355 is relatively limited. Nylund et al. noted that stable LPA results usually demand larger samples, particularly for small subgroups. While our two-profile model yielded an acceptable Entropy (>0.80), the classification findings should be interpreted with caution. Future large-sample multicenter studies are required to replicate these latent profiles. Fourth, cross-sectional studies cannot determine causal relationships between mindful self-care and professional grief. Future research could expand the sample collection scope or conduct longitudinal studies to further validate the findings.

## Conclusion

This study used latent profile analysis to categorize nurses' mindful self-care into two latent profiles: “Mindful Self-Care Deficient Profile” and “High Mindfulness Care Profile.” ICU nurses over 30 years old, with good health status and high self-compassion are more likely to develop into the high mindfulness care profile. Nurses with high mindfulness care profile have lower levels of professional grief. These findings can provide some basis for improving mindful self-care and professional grief among ICU nurses.

## Data Availability

The original contributions presented in the study are included in the article/supplementary material, further inquiries can be directed to the corresponding author.
